# Prevalence, Virulence, and Antimicrobial Resistance of *Campylobacter* spp. in Raw Milk, Beef, and Pork Meat in Northern Poland

**DOI:** 10.3390/foods8090420

**Published:** 2019-09-17

**Authors:** Małgorzata Andrzejewska, Bernadeta Szczepańska, Dorota Śpica, Jacek J. Klawe

**Affiliations:** Department of Hygiene, Epidemiology and Ergonomics Nicolaus Copernicus University in Toruń and Ludwik Rydygier Collegium Medicum in Bydgoszcz, 9 Marii Curie Skłodowskiej St., 85-094 Bydgoszcz, Poland; b.szczepanska@cm.umk.pl (B.S.); dorota.spica@cm.umk.pl (D.Ś.); jklawe@cm.umk.pl (J.J.K.)

**Keywords:** *Campylobacter* spp., beef, pork, unpasteurized milk, antimicrobial resistance, virulence genes

## Abstract

The purpose of this study was to determine whether raw milk, unpasteurized dairy products, pork, and beef available for sale in the Kujawsko-Pomorskie and Wielkopolska regions in Poland are contaminated with *Campylobacter* spp. bacteria and may be a potential source of infection. For isolated strains, antibiotic susceptibility and the presence of genes responsible for virulence were examined. Material for research included 1058 food samples collected between 2014 and 2018 with 454 samples of raw milk and unpasteurized dairy products (milk from vending machines, milk from owners of dairy cows, cheese, milk cream) and 604 samples of raw meat (pork, beef). The results indicated that 9.3% of the samples were positive for *Campylobacter* spp., and *Campylobacter* *jejuni* was predominant in this study. *Campylobacter* bacteria was not found in milk collected from vending machines, as well as cheese and milk cream samples. *Campylobacter* was noted in 12.7% of beef samples, 11.8% of raw milk purchased from individual suppliers, and 10.9% of pork samples. Resistance to erythromycin (2.0%), azithromycin (3.1%), gentamicin (4.1%), tetracycline (65.3%), and ciprofloxacin (71.4%) was determined using the disc diffusion method. Furthermore, the prevalence of *racR*, *sodB*, *csrA*, *virB11*, *cdtB*, *iam*, and *wlaN* genes were examined using the PCR method. The *sodB*, *csrA*, and *cdtB* genes exhibited the highest detection rate, but none of the genes were identified in 100% of the isolates. Statistically significant differences between the presence of virulence marker genes, including for *iam*, *racR*, and *csrA* markers, were noted among different sources of the isolates. Differences in the distribution of *iam,* *wlaN*, and *virB11* were also shown between *C. jejuni* and *C. coli* strains. As a result of the analysis, it has been concluded that unpasteurized milk, beef, and pork could be a sources of *Campylobacter* pathogens. Moreover, this study revealed virulent properties of *Campylobacter* isolated from such food products and high resistance rates to fluoroquinolones, which may represent difficulties in campylobacteriosis treatment.

## 1. Introduction

Increased interest in the *Campylobacter* bacteria has been observed for more than 30 years [1,2,3]. Campylobacteriosis is an infectious disease caused in humans by the bacteria belonging to the *Campylobacter* genus, especially *C. jejuni* and *C. coli*, and is the primary cause of human bacterial gastroenteritis worldwide [4]. *Campylobacter* organisms are currently the most common factor of foodborne infections caused by food intake, especially foods of animal origin [5,6]. Data from the European food safety authority (EFSA) show that approximately 200 thousand people per year in the countries of the European Union suffer from food infections caused by *Campylobacter* [7].

*Campylobacter* bacteria are widespread in nature. The natural reservoir of *Campylobacter* is the digestive tract of birds (as a component of natural flora) and other domesticated and free-living animals [8,9]. The presence of bacteria in livestock is associated with the contamination of food products of animal origin. Meat obtained from poultry is the most common source of *Campylobacter* bacteria, but pork, beef, and unpasteurized milk also represent sources of infection with these microorganisms [4,10,11,12].

Pork and beef have long been preferred in many countries, and their level of consumption depends on the availability of the product on the market. The presence of *Campylobacter* in cattle and pig carcasses at slaughterhouse level is well documented and significant [13,14]. However, the occurrence of *Campylobacter* in beef or pork is lower than that in poultry meat [15]. A lower rate of *Campylobacter* isolation from beef or pork meat can be associated with longer slaughter time, cooling of carcasses, and drying of the meat surface [16].

Raw (unpasteurized) milk has been linked to many foodborne illnesses, including *Campylobacter* infections. Campylobacteriosis cases caused by ingestion of unpasteurized milk have been recorded worldwide [17,18]. In the USA, the number of outbreaks associated with unpasteurized milk clearly increased in a 6-year study [19]. An estimated 3% of the U.S. population drinks raw milk and prefers it to pasteurized milk, given perceived health benefits [18].

Raw milk and unpasteurized milk products are currently sold in Poland. Customers can buy such products directly from farmers or vending machines. Such machines can be found in most major cities. Although vending machines are subject to strict hygiene standards, there are reports on the prevalence of *Campylobacter* in raw milk from such devices [20,21].

Raw milk can be contaminated with the *Campylobacter* in a variety of ways. *Campylobacter* bacteria are ubiquitous in cattle and dairy farms. Poorly cleaned machines, bovine diseases (*mastitis*), and, most often, fecal contamination of the milk from the known reservoir represent documented causes of milk pollution during the reported outbreaks of *Campylobacter* infection [18,19,20,21,22].

Resistance to antimicrobial substances among zoonotic bacteria is currently the subject of particularly intensive research concerning the entire food chain, given the importance of this phenomenon in public health. Infections caused by drug-resistant strains require a long treatment duration, have a higher morbidity and mortality, and are associated with higher treatment costs. In recent years, a significant increase in resistance among *Campylobacter* bacteria has been noted [7,23]. This problem applies to the strains isolated from humans, animals, and food. One of the main reasons for this phenomenon is the excessive use of antibiotics [12,24,25]. To estimate antibiotic resistance profile in *Campylobacter* isolates, we choose: azithromycin, erythromycin, gentamicin, ciprofloxacin, and tetracycline, antimicrobials clinically used and most often tested in both food/animal and human isolates. Macrolides (e.g., erythromycin) and fluoroquinolones (e.g., ciprofloxacin) are considered the drugs of choice. Other antibiotics such as gentamicin, tetracycline, and azithromycin are listed as alternative drugs for the treatment of systemic *Campylobacter* infections [7,23,24,25].

Although primarily associated with self-limiting acute enteritis, *Campylobacter* infections can be invasive and lead to long-term complications, including Guillain-Barré syndrome [26]. Virulence factors affecting the pathogenicity of these microorganisms are not completely understood [27,28]. The basis of *Campylobacter* spp. pathogenicity involves a set of mechanisms, including motility and the ability to adhere to the enterocytes in the process of adhesion and penetration of host cells (invasion). Other confirmed bacterial virulence factors include toxin production and protection against oxidative stress [29,30,31,32]. A notable complication of campylobacteriosis infection is the development of the Guillain-Barré syndrome related to *C. jejuni* sialylated lipooligosaccharides (LOS) that exhibit molecular mimicry with gangliosides on peripheral nerves [27].

In the present study, seven genes that are important in the pathogenesis of campylobacteriosis were chosen on the basis of previously published data. The *racR* gene participates in adhesion and colonization. The *cdtB* gene is involved in cytotoxin production. The *iam* sequence is probably connected with a diarrhoeal form of the disease [29,33]. Another virulence gene linked with *Campylobacter* spp. is the invasion-associated marker *virB11*. *WlaN* was selected because it is involved in the expression of ganglioside mimics in Guillain–Barré syndrome. The *sodB* and *csrA* genes provide protection against oxidative stress [30,34].

The purpose of this study was to determine whether raw milk, unpasteurized dairy products, pork, and beef available for sale on the market in two regions in Northern Poland are contaminated with *Campylobacter* spp. and may be a potential source of infection. We also compared the occurrence of *Campylobacter* spp. pathogenic genes responsible for encoding virulence factors and antibiotic susceptibility in *Campylobacter jejuni* and *Campylobacter coli* positive samples isolated from different origins.

## 2. Materials and Methods

### 2.1. Sample Collection

A total of 1058 food samples of animal origin were collected in Northern Poland over a four-year period (2014–2018). Materials for the study include 454 samples of raw milk and unpasteurized dairy products (cheese, cream) originating from Kujawsko-Pomorskie and Wielkopolska regions. Samples were purchased year-round directly from the owners of the dairy cows (*n* = 221), automatic vending machines (*n* = 113), and local markets (*n* = 120). We examined 604 samples of raw meat samples that were sold unpackaged (beef *n* = 347 and pork *n* = 257). The samples were purchased year-round from supermarkets and butcher shops located in the study area to provide the element of representativeness. The number of samples collected during each year of the study was as followed: October 2014/September 2015—260 food samples, October 2015/September 2016—270 food samples, October 2016/September 2017—273 food samples, October 2017/September 2018—255. Table 1 presents all samples collected and tested over the study period. The samples were transported to the laboratory in cooler boxes on ice and analyzed immediately.

### 2.2. Isolation of Campylobacter 

Isolation of *Campylobacter* spp. from meat and milk was conducted in compliance with the EN ISO 10272-1:2006 method with slight modification. Briefly, meat and cheese (25 g) were homogenized for 2 min in a stomacher with 225 mL buffered peptone water (Oxoid Limited, Basingstoke, United Kingdom) in the sterile plastic bags. Then, 10 mL of the homogenate solution and 10 ml of raw milk was added to 90 mL of Bolton broth containing the Bolton broth selective supplement and 5% laked horse blood (Oxoid Limited, Basingstoke, United Kingdom) and incubated at 42 °C for 48 h under microaerobic conditions (Generbox microaer-BioMerieux, Marcy l’Etoile, France). Next, the bacterial suspension was spread onto CCDA plates (Oxoid Limited, Basingstoke, United Kingdom) and then incubated at 42 °C for 48 h under microaerobic conditions. Characteristic growth from the CCDA plates was transferred to a blood plate (Columbia agar containing 5% cattle blood, Oxoid Limited, Basingstoke, United Kingdom) and incubated overnight at 42 °C. Colonies suspected as *Campylobacter* spp. were examined for cell morphology, motility, and oxidase reactions. Putative *Campylobacter* colonies were frozen at −80 in Microbanks (Pro-Lab Diagnostics, Birkenhead, United Kingdom) until species differentiation using polymerase chain reaction (PCR).

### 2.3. Species Identification

The PCR method with specific primers, as previously described, was used for the purpose of identification of colonies as *C. jejuni* or *C. coli* [35,36]. Bacterial DNA lysates were prepared from fresh Campylobacter cultures using the boiling method, as previously described [37]. PCR was performed in a 25 mL volume containing 2.5 mL of 10× PCR buffer (Thermo Fisher Scientific, Waltham, MA, US), 2.5 mL of 25 mM MgCl_2_ (Thermo Fisher Scientific, Waltham, MA, US), 1.0 mL of each PCR primer (10 mM—Institute of Biochemistry and Biophysics Polish Academy of Sciences, Warsaw, Poland ), 1.0 mL of 10 mM dNTP mix (Thermo Fisher Scientific, Waltham, MA, US), 0.5 mL of Dream Taq DNA Polymerase (1 U/mL—Thermo Fisher Scientific, Waltham, MA, US), 1.0 mL of template, and 13.0 mL of DNA-free purified water (Thermo Fisher Scientific, Waltham, MA, US). PCR was performed using the cycling conditions specified by the original authors [35,36]. The amplified DNAs were analyzed by electrophoresis in a 1.5% agarose gel. Reference strains of *C. jejuni* (NCTC11322) and *C. coli* (NCTC11366) were used as a control strain.

### 2.4. Prevalence of Virulence Genes

The presence of *racR*, *sodB*, *csrA*, *virB11*, *cdtB*, *iam*, and *wlaN* genes was determined using the PCR method with the primers and cycling conditions, as previously described by Linton et al., 2000 (for *wlaN*); Bang, Scheutz and Ahrens, 2001 (for *cdtB*); Carvalho et al., 2001 (for *iam*); Datta, Niwa, and Itoh, 2003 (for *racR*, *virB11*); Fields and Thompson, 2008 (for *csrA*) and Biswas et al., 2011 (for *sodB*) [29,30,34,38,39,40], Supplementary Table S1. All PCRs were performed in 25 μL volume reactions containing 2.5 μL of 10× PCR buffer (Thermo Fisher Scientific, Waltham, Ma, US), 2.5 μL of MgCl_2_ (25 mM, Thermo Fisher Scientific, Waltham, MA, US) 1.0 μL of each PCR primer (10 μM, Institute of Biochemistry and Biophysics Polish Academy of Sciences, Warsaw, Poland), 0.5 μL of deoxynucleoside triphosphate mix (10 mM, Thermo Fisher Scientific, Waltham, MA, US), 0.5 μL of Dream Taq DNA Polymerase (0.5 U/μL, Thermo Fisher Scientific, Waltham, Ma, US), 2.0 μL of template, and 15.0 μL of DNA-free purified water (Thermo Fisher Scientific, Waltham, MA, US). Visualization of DNA paths was obtained by adding the Midori Green DNA Stain (Nippon Genetics, Duren, Germany) to 1% agar gel prior to electrophoresis. The size of the amplicon was compared using a 100-bp DNA size marker (Thermo Fisher Scientific, Waltham, MA, US).

### 2.5. Antimicrobial Resistance

Susceptibility to five antimicrobial agents was assessed by the disk diffusion method according to the clinical laboratory and standard institute [41] using Mueller-Hinton medium supplemented with 5% defibrinated horse blood (Oxoid, Basingstoke, United Kingdom). The antimicrobials tested included ciprofloxacin 5 µg, erythromycin 15 µg, gentamicin 10 µg, tetracycline 30 µg, and azithromycin 15 µg (Oxoid, Basingstoke, United Kingdom). In the cases when CLSI recommendations were not available for Campylobacters, CLSI guidelines for Enterobacteriaceae were followed. The strains that showed resistance to three or more classes of antimicrobial agents were considered multidrug-resistant (MDR) strains. Reference strains of *C. jejuni* (NCTC11322) and *C. coli* (NCTC11366) were used as a control strain.

### 2.6. Statistical Analysis

Statistical analysis was performed using the Statistica 10.0 program (StatSoft, Cracow, Poland, 2011). Statistical differences in the prevalence of virulence genes and antimicrobial resistance between individual samples and between *C. jejuni* and *C. coli* strains were analyzed using the Chi square test. Holm–Bonferroni correction was applied for multiple comparisons. For small sample sizes, Yates’ correction was also used. A significance level of *p* = 0.05 was accepted. *p*-values of < 0.05 were considered statistically significant.

## 3. Results

### 3.1. Prevalence of Campylobacter spp. in Milk, Pork, and Beef

During our 4-year study, 1058 samples from dairy products, fresh pork, and beef meat were analyzed (Table 1). The results have indicated the presence of *Campylobacter* spp. in 98 (9.3%) of the samples. Frequency of *C. jejuni* in the examined samples was 76.5%, *C. coli* was found in 23.5% of the analyzed samples. The highest prevalence of *Campylobacter* spp. (12.7%) was noted in beef samples followed by raw milk from farmers (11.8%). Sample contamination with *Campylobacter* bacteria was the lowest in the pork meat group (10.9%). The occurrence of *Campylobacter* in the samples obtained from pork chops was slightly higher than in minced pork samples. Not all types of food exhibited *Campylobacter* spp. contamination. Raw, unpasteurized milk obtained from vending machines, as well as cheese and milk cream purchased from retail stores, were not contaminated with *Campylobacter* bacteria. *Campylobacter* isolates recovered from milk exclusively included *C. jejuni*, while both species were detected at the same frequency (50%) in pork meat products. The Chi square test revealed that *C. jejuni* isolates were significantly more frequently isolated than *C. coli* isolates in beef meat samples (*p* < 0.5).

### 3.2. Prevalence of Virulence Genes

Overall, the occurrence of virulence markers varied among different sources of the isolates, and significant differences were found for genes associated with invasiveness (*iam*), adherence, colonization (*racR*), and stress response (*csrA*). Overall, the most common virulence markers were *sodB* (90.8%), *cdtB* (88.8%), and *csrA* (71.4%) genes, which are associated with stress response and toxin production (Table 2).

High levels of *C. jejuni* virulence genes were noted in milk, with the exception of *racR, wlaN*, and *virB11* genes, which were present at relatively low levels. Among all *Campylobacter* spp. isolates obtained from beef, genes responsible for toxin production, stress response, and colonization were the most common. The majority of genes were found at high levels in *Campylobacter* spp. isolated from pork meat (*csrA*, *sodB, cdtB*, and *racR*). Low levels of the pathogenic markers *virB11* and *iam* were noted in *Campylobacter* isolates from pork and beef meat. Significant differences in the occurrence of *iam*, *wlaN*, and *virB11* genes were detected between *C. jejuni* and *C. coli* isolates (Table 3).

### 3.3. Antimicrobial Resistance

The results for antimicrobial resistance in relation to sample origin (milk, pork, beef) and species (*C. jejuni* or *C. coli*) are shown in Table 4 and Table 5. Overall, most of the strains were resistant to ciprofloxacin and tetracycline. Ciprofloxacin is one of the two antimicrobials regarded as critically important for treatment of *Campylobacter* infections in humans, and very high (71.4%) resistance levels were reported in all *Campylobacter* isolates. Resistance to tetracycline was confirmed in 65.3% of the strains. The lowest antimicrobial resistance rates were noted for erythromycin, azithromycin, and gentamicin (2.0%, 3.1%, and 4.1%, respectively).

Tetracycline resistance was the most common in *Campylobacter* stains isolated from milk (77.0%). Ciprofloxacin and gentamicin resistance rates were estimated as 65.4% and 3.8%, respectively. All isolates from milk were susceptible to azithromycin and erythromycin.

Resistance to ciprofloxacin was the most common in isolates from beef meat (63.4%) followed by tetracycline (59.1%). *Campylobacter* isolates obtained from beef (2.3%) exhibited the lowest antimicrobial resistance rates to erythromycin and azithromycin.

Among the identified *C. coli* strains from pork samples, no strains sensitive to ciprofloxacin were obtained. In total, 64.3% of strains obtained from pork were resistant to tetracycline. Resistance to gentamicin was detected in three *Campylobacter* pork isolates (10.7%), and resistance to azithromycin and erythromycin was estimated as 7.2% and 3.6%, respectively. Significant differences in *Campylobacter* ciprofloxacin resistance were noted between *C. coli* isolated from pork and beef samples. One strain of *C. coli* obtained from a pork sample presented simultaneous resistance to ciprofloxacin, erythromycin, and azithromycin.

## 4. Discussion

Most of the available studies are mainly concerned with the prevalence of *Campylobacter* in poultry as a main source of human campylobacteriosis. The number of studies discussing *Campylobacter* contamination in other meat types is limited in the literature. In our study, we emphasized that *Campylobacter* contamination in retail dairy and meat products other than poultry also raise concern, especially given the high resistance profile of milk, beef, and pork *Campylobacter* isolates.

In this study, 28 out of 257 (10.9%) of pork meat samples were positive for *Campylobacter* spp., which corroborates findings reported in Poland [15]. Other reports on the prevalence of *Campylobacter* spp. in retail pork meat revealed lower contamination (1.7%—Zhao et al., 2001; 1.6%—Hong et al., 2007; 2%—Noormohamed and Fakhr, 2013; 0.5%—Lappiere et al., 2016) [42,43,44,45]. Pork and beef samples were negative for *Campylobacter* in a Canadian study [16]. These differences may be due to several variables, including geographical location, different sanitary conditions on farms, or slaughter practices. Most of the authors indicate that the main species isolated from pigs or retail pork products was *C. coli,* and a similar result is reported in our study [10,42].

The prevalence of *Campylobacter* spp. in retail beef meat products in our study is similar to previous research in Poland that reported *Campylobacter* contamination in 10.1% of fresh beef [15]. Similar levels of contamination were revealed in studies of Kashoma et al., 2016—9.5%, and in Pakistan, where the occurrence of *Campylobacter* was estimated in 15.5% of beef by Nisar et al., 2018 [10,46]. *Campylobacter* was present in 78% of beef livers in Iran [44]. Previous studies in Poland reported that 10% and 30% of bovine and pig carcasses, respectively, were positive for *Campylobacter* spp. [13].

Recent studies clearly indicate that the pork and beef may be contaminated with *Campylobacter* and constitute a potential source of campylobacteriosis infection in humans. To protect consumers, there is a need for greater realization of food safety programs "from the farm to the consumer", further risk assessment, and consumer education.

*Campylobacter* spp. was isolated from 11.8% of retail raw milk samples in this study, which is similar to levels obtained by Hussaina et al., 2007—10.2%, Kashoma et al., 2016—13.4%, and Raeisi et al., 2017—8.7% [10,12,47]. A lower rate of *Campylobacter* prevalence in raw milk samples was previously reported in Poland in the study by Wysok, Wiszniewska, Uradziński, and Szteyn, 2011 [22]. In our study, no bacteria was isolated from vending machines. Despite a relatively low prevalence of *Campylobacter* spp. in retail raw milk samples, many authors suggest that raw milk could represent the second most common source of human campylobacteriosis, after chicken meat [10,22,48]. Organic and raw food is becoming increasingly popular, given their natural health properties, but consumers should also be aware of the risk associated with consuming unpasteurized milk [49]. Consumption of raw milk is inherently risky because the milk has not been treated to inactivate pathogens.

Data on the occurrence of *Campylobacter* virulence markers in food samples are mainly related to the detection of virulence genes in *Campylobacter* isolated from poultry. The number of studies on the prevalence of genes related to virulence among isolates of *Campylobacter* spp. from milk, beef, and pork is limited. In this study, among the 98 *Campylobacter* strains, the most frequently detected genes were *sodB*, *cdtB*, and *csrA* genes. Research conducted by Modi, Brahmbhatt, Chatur, and Nayak (2015) showed that the *cdtB* gene (involved in toxin production) was detected in all *C. jejuni* isolates from raw milk samples [48]. Similarly, the increased prevalence of the *cdtB* gene was demonstrated in *C. jejuni* isolates obtained from pork liver [50]. The low prevalence of the *cdtB* gene in *C. coli* stains (5.9%) in the same study is noteworthy. The *cdtB* gene was noted in 60% of *Campylobacter* isolates from chicken, pork, and turkey meat samples in studies performed by Lapierre et al. [45].

The available literature provides a few works on the detection of *sodB* and *csrA* genes in milk, pork, or beef in *Campylobacter* isolates [27,51]. Most of the authors underline their crucial role in campylobacteriosis pathogenesis. *SodB* exhibited a high prevalence among poultry meat and human *Campylobacter* strains analyzed in the study of Wieczorek, Wołkowicz, and Osek, 2018 [51]. The *csrA* gene was present in 87.3% of *C. jejuni* isolates form broiler meat and in 97.7% from bovine in the studies of Gonzalez-Hein, Huaracán, García, and Figueroa, 2013 [27]. The authors suggested an important regulatory role of this gene in the stress responses in *C. jejuni* pathogenesis. Our study revealed significant differences in the distribution of *csrA* genes between different *Campylobacter* sources.

In the current study, *racR*, which was selected as pathogenic gene responsible for adherence and colonization, was statistically detected more often in pork or beef *Campylobacter* isolates than those recovered from milk. Similar to the results of this study, Bardon et al. detected *racA* at high frequencies in *Campylobacter* pork liver samples [50]. Greater than 90% of *C. jejuni* isolates from chicken harbor the *racR* gene in the studies of Datta. Niwa and Itoh, 2003 [40]. Moreover, Wysok and Wojtacka, 2018, reported a high prevalence of the *racR* gene in isolates obtained from cattle and swine [14].

The *iam* gene is a virulence marker examined in the present study that determines the invasiveness of *Campylobacter* spp. The prevalence of *iam* differed between both species and sources. The *iam* gene was present in 84.6%, 35.7%, and 25% of *Campylobacter* obtained from milk, pork, and beef, respectively. A high frequency of this invasion marker was previously detected in *Campylobacter* spp. from food samples, as reported by Rizal et al., 2010 [52]. On the other hand, Modi, Brahmbhatt, Chatur, and Nayak (2015) identified the *iam* gene in 14.3% of *C. jejuni* isolates from milk [48]. In the study by Bardon et al. (2017), the *iam* sequence was exclusively found in *C. coli* isolates from pork liver, which is consistent with the present study wherein *iam* was primarily detected in the *C. coli* group [50]. Given that the *iam* sequence appeared at different frequencies between different species and sources, it can be concluded that the role of this marker in the pathogenesis of *Campylobacter* spp. infections is unclear. Further studies need to be performed to explain the causes of such differentiation.

*WlaN* was selected as a pathogenic gene responsible for the ganglioside mimicking Guillain–Barré syndrome. A limited number of studies have reported *wlaN* gene detection in *Campylobacter* obtained from meat or milk. The occurrence of this gene in *Campylobacter* strains from pork, beef, and milk was estimated at approximately 50% in this study, and significant differences were between *C. jejuni* and *C. coli* strains (*p* < 0.05). Raeisi et al. (2017) described *wlaN* detection exclusively in *C. coli* isolates recovered from raw milk samples [12]. In the study by Lapierre et al. (2016), *C. jejuni* strains isolated from meat showed increased *wlaN* gene frequency compared with *C. coli*, which was also observed in *C. jejuni* isolates from pork in our study [45].

The least prevalent gene in our study was *virB11* (20.4%). This gene was a statistically more often detected gene in *C. coli* isolates. The plasmid-associated virulence marker *virB11* was not detected in isolates from milk in Raeisi et al.’s studies [12]. In Bardon et al.’s study (2017), the *virB11* gene was only confirmed in *C. coli* isolates obtained from pork liver with a low detection rate—5.9% [50].

Resistance to antimicrobials in *Campylobacter* spp. isolated from beef and pork has not previously been examined in Poland. Data on antibiotic profiling showed that *Campylobacter* isolated from pig and cattle carcasses at slaughter in Poland were most frequently resistant to quinolones—57.1% and tetracycline—51.4% [53]. The prevalence of *Campylobacter* isolated from red meat being highly resistant to ciprofloxacin and tetracycline (88.8% and 66.7%, respectively) was confirmed by Raeisi et al. [12]. Lapierre et al. (2016) demonstrated that all *C. coli* strains from pork liver were resistant to ciprofloxacin and tetracycline, whereas *C. jejuni* isolated from bovine meat were sensitive to ciprofloxacin [45]. According to an EFSA report (2019) in 2017, more than half of the tested *Campylobacter* isolates from either pigs or cattle exhibited resistance to fluoroquinolones. Considering that the *C. coli* isolates from pig meat were tested for susceptibility in 2016, resistance to ciprofloxacin (76.5%) and tetracycline (85.3) was very high. Increasing trend of resistance to tetracycline in *C. coli* isolated from pig meat was observed in Portugal (66.7% in 2016–100% in 2017) [7].

Raw milk *C. jejuni* isolates in our study were also highly resistant to ciprofloxacin and tetracycline. Increased resistance of *Campylobacter* from raw milk to this antimicrobial agent (85.8%) was previously recorded in Poland [22]. Similar data regarding the isolation of *Campylobacter species* from milk that were highly resistant to ciprofloxacin and tetracycline (greater than 85%) were reported by Modi, Brahmbhatt, Chatur, and Nayak, 2015 [48]. In contrast, Kashoma et al. (2016) reported that only 9.3% and 11.8% (depending on the method) of *Campylobacter* isolated from milk samples and beef carcasses, respectively, exhibiting resistance to ciprofloxacin [10]. Similar rates for ciprofloxacin resistance were detected in the US [54]. An outbreak of fluoroquinolone-resistant *C. jejuni* infections associated with raw milk consumption in United States of America was recently described by Burakoff et al., 2018 [55].

Reports from all over the world recently noted emerging resistance to macrolides in clinical, environmental, animal, and food *Campylobacter* samples [7,56,57]. *Campylobacter* isolates in our study were mostly sensitive to the optimal antibiotics used for *Campylobacter* infection (especially in children), but we confirmed single resistance for macrolides and gentamicin, which was not previously described in *Campylobacter* isolated from pork, beef, or milk in Poland. Noteworthy is the increasing trend in gentamicin resistance in *C. jejuni* and *C. coli* isolates from broilers, cattle, pigs, and their products, reported by EFSA in 2017 (percentages for gentamicin resistance in *C. jejuni* from cattle and *C. coli* from pigs were higher than 65% in Croatia in 2017) [7].

The isolation of *Campylobacter* bacteria resistant to azithromycin in raw milk was previously described by Kashoma et al. [10]. The percentage of strains resistant to azithromycin and erythromycin was significant in a study by Noormohamed and Fakhr (2013), especially in *C. coli* recovered from beef liver samples [40]. In contrast, Raeisi et al. (2017) reported that *Campylobacter* recovered from milk and cattle samples were sensitive to erythromycin [12].

One *C. coli strain* isolated from a pork sample in our study was resistant to three antibiotics simultaneously and expressed putative virulence genes. These data raise public health concerns, especially given that antimicrobial resistance was observed for the drug of choice for clinical treatment of *campylobacteriosis.* Multi-drug resistant patterns were previously described for milk and beef *Campylobacter* isolates in the studies of Kashoma et al., 2016, and Raeisi et al., 2017 [10,12].

## 5. Conclusions

We reported on *Campylobacter* contamination of unpasteurized milk, pork, and beef sold in retail in Poland that may represent potential sources of infection. High resistance rates for fluoroquinolones, and emergence of MDR isolates from pork sample are reported. Moreover, a high level of resistance to ciprofloxacin and tetracycline among *C. jejuni* and *C. coli* species indicate the reduced clinical utility of these antibiotics for the treatment of patients. There is also a need for further monitoring of food products in relation to possible transmission of resistant *Campylobacter* to humans. The present study is the first in Poland to assess the frequency of genes responsible for virulence at different stages of pathogenesis among strains of *Campylobacter* isolated from food of animal origin, such as milk, beef, and pork. In this study, the number of strains with the key virulence factors was significant; however, differences in the frequency of genes between different sources and species of *Campylobacter* were also described, which should be further verified.

## Figures and Tables

**Table 1:** Prevalence of *Campylobacter* isolates from different sources in Poland.

Sample Type	No. of Samples Tested	No. (%) of Samples Positive for *Campylobacter*	No. (%) of Samples Positive for *C. jejuni*	No. (%) of Samples Positive for *C. coli*
Dairy products - raw milk (from vending machines) - raw milk (farmers) - cheese from unpasteurized milk - milk cream - total	113 221 60 60 454	0 26 (11.8) 0 0 26 (5.7)	0 26 (100) 0 0 26 (100)	0 0 0 0 0
Beef meat - beef cuts	347	44 (12.7)	35 (79.5)	9 (20.5)
Pork meat - pork chops - minced meat - total	151 106 257	19 (12.6) 9 (8.4) 28 (10.9)	9 (47.4) 5 (55.5) 14 (50)	10 (52.6) 4 (44.4) 14 (50)
Total	1058	98 (9.3)	75 (76.5)	23 (23.5)

**Table 2:** Distribution of virulence genes in *Campylobacter* spp. isolated from different sources.

Origin No. of Isolates				*Campylobacter* Virulence Factor No. of Isolates with The Occurrence of The Gene (%)					
			iam *	racR *	*wlaN*	*cdtB*	*sod B*	csrA *	*virB11*
Milk	Total	26	22 (84.6)	7 (26.9)	13 (50.0)	24 (92.3)	24 (92.3)	15 (57.7)	4 (15.4)
	*C. jejuni*	26	22 (84.6)	7 (26.9)	13 (50.0)	24 (92.3)	24 (92.3)	15 (57.7)	4 (15.4)
Beef meat	Total	44	11 (25)	29 (66.0)	18 (40.9)	40 (90.9)	39 (88.4)	29 (65.9)	9 (20.5)
	*C. jejuni*	35	2 (5.7)	25 (71.4)	14 (40.0)	34 (97.1)	33 (94.3)	22 (62.9)	6 (17.1)
	*C. coli*	9	9 (100)	4 (44.4)	4 (44.4)	6 (66.6)	6 (66.6)	8 (88.8)	3 (33.3)
Pork meat	Total	28	10 (35.7)	18 (64.3)	14 (50.0)	23 (82.1)	25 (89.3)	26 (92.9)	7 (25.0)
	*C. jejuni*	14	2 (14.3)	10 (71.4)	14 (100.0)	11 (78.6)	13 (92.9)	13 (92.9)	5 (35.7)
	*C. coli*	14	8 (57.1)	8 (57.1)	0	12 (85.7)	12 (85.7)	13 (92.8)	2 (14.3)
	Total	98	43 (43.9)	54 (56.8)	45 (45.9)	87 (88.8)	89 (90.8)	70 (71.4)	20 (20.4)

* Statistically significant differences between the presences of virulence marker genes among different sources of the isolates have been identified. The following differences were identified for the presence of *iam* from milk and beef meat (*p* < 0.0000), *iam* from milk and pork meat (*p* = 0.0014), *racR* from milk and beef meat (*p* = 0.0048), racR from milk and pork meat (*p* = 0.0118), *csrA* from milk and pork meat (*p* = 0.0207), and *csrA* from pork and beef meat (*p* = 0.0207).

**Table 3:** Detection of virulence genes in *C. jejuni* and *C. coli* strains (%).

Species	No. of Isolates	*iam*	*wlaN*	*racR*	*cdtB*	*sod B*	*csrA*	*virB11*
*C. jejuni*	*n* = 75	26 (34.6)	41 (54.6)	42 (56.0)	69 (92.0)	70 (93.3)	50 (66.6)	5 (6.6)
*C. coli*	*n* = 23	17 (73.9)	4 (17.4)	12 (52.2)	18 (78.3)	19 (82.6)	20 (87.0)	15 (65.2)
*p*		0.0009	0.0017	0.7469	0.0679	0.2521	0.0595	0.0000

**Table 4:** Antimicrobial resistance of *C. jejuni* and *C. coli* isolated from different sources.

Antimicrobial Agent	*Campylobacter* Species	No. of Resistant Isolates/ No. of Isolates (%)		
		Milk	Beef Meat	Pork Meat
Erythromycin	*C. jejuni* *C. coli* *Total*	0/26 0/0 0/26	1/35 (2.9) 0/9 1/44 (2.3)	0/14 1/14 (7.1) 1/28 (3.6)
Azithromycin	*C. jejuni* *C. coli* *Total*	0/26 0/0 0/26	1/35 (2.9) 0/9 1/44 (2.3)	1/14 (7.1) 1/14 (7.1) 2/28 (7.2)
Ciprofloxacin	*C. jejuni* *C. coli* *Total*	17/26 (65.4) 0/0 17/26 (65.4)	22/35 (62.9) 6/9 (66.7) * 28/44 (63.4)	11/14 (78.6) 14/14 (100.0) * 25/28 (89.3)
Tetracycline	*C. jejuni* *C. coli* *Total*	20/26 (77.0) 0/0 20/26 (77.0)	21/35 (60.0) 5/9 (55.5) 26/44 (59.1)	10/14 (71.4) 8/14 (57.1) 18/28 (64.3)
Gentamicin	*C. jejuni* *C. coli* *Total*	1/26 (3.8) 0/0 1/26 (3.8)	0/35 0/9 0/44	2/14 (14.2) 1/14 (7.1) 3/28 (10.7)

* Statistically significant differences between antimicrobial resistance to ciprofloxacin among *C. coli* isolates from beef and pork were detected (*p* = 0.0407).

**Table 5:** Antimicrobial resistance in *C. jejuni* and *C. coli* isolates.

Species	No. of Isolates	ERY	AZT	CIP	TET	GEN
		No. of Resistant Isolates (%)				
*C. jejuni*	*n* = 75	1 (1.3)	2 (2.6)	50 (66.7)	51 (68.0)	3 (4.0)
*C. coli*	*n* = 23	1 (4.3)	1 (4.3)	20 (87.0)	13 (56.5)	1 (4.3)
*C. jejuni + C. coli*	*n* = 98	2 (2.0)	3 (3.1)	70 (71.4)	64 (65.3)	4 (4.1)
*p*		0.9588	0.7777	0.0595	0.3117	0.9121

## Supplementary Materials

The following are available online at https://www.mdpi.com/2304-8158/8/9/420/s1, Table S1: PCR primers used for detection of campylobacter virulence genes.
