# Supplementary material for: Prevalence, Virulence, and Antimicrobial Resistance of Campylobacter spp. in Raw Milk, Beef, and Pork Meat in Northern Poland
Source: Foods. 2019 Sep 17;8(9):420. doi: 10.3390/foods8090420 (PMC6770586; doi:10.3390/foods8090420)
Supplement: Supplementary file 1 [file foods-08-00420-s001.pdf]

Supplementary Table 1. PCR primers used for detection of *Campylobacter* virulence genes.

Primers	Sequence (5′ → 3′)	Product (bp)	Annealing Temperature	References
racR-F<br>*racR*-R	GATGATCCTGACTTTG<br>TCTCCTATTTTTACCC	584	45 ˚C	Datta et al., 2003
virB11-F<br>*virB11*-R	TCTTGTGAGTTGCCTTACCCCTTTT<br>CCTGCGTGTCCTGTGTTATTTACCC	494	53 ˚C	Datta et al., 2003
cdtB-F<br>*cdtB*-R	GTTAAAATCCCCTGCTATCAACCA<br>GTTGGCACTTGGAATTTGCAAGGC	495	42 ˚C	Bang et al., 2001
Iam-F<br>*Iam*-R	GCGCAAAATATTATCACCC<br>TTCACGACTACTATGCGG	518	52 ˚C	Carvalho et al., 2004
wlaN-F<br>*wlaN*-R	TTAAGAGCAAGATATGAAGGTG<br>CCATTTGAATTGATATTTTTG	672	46 ˚C	Linton et al., 2000
sodB-F<br>*sodB*-R	ATGATACCAATGCTTTTGGTGATTT<br>TAATACGACTCACTATAGGGCATTTGCATAAAAGCTAACTGATCC	638	50 ˚C	Biswas et al., 2011
csrA-F<br>*csrA*-R	CACAGTCAGTGAAGGTGCTT<br>ACTCGCACAATCGCTACTTC	878	58 ˚C	Fields & Thompson, 2008
